# Successful pregnancy outcome after sonographic control and gasless laparoscopic removal of 810-gram fibroid during pregnancy: case report

**DOI:** 10.52054/FVVO.16.3.035

**Published:** 2024-09-30

**Authors:** E Piriyev, T Römer

**Affiliations:** University Witten-Herdecke, Germany; University of Cologne, Germany; Department of Obstetrics and Gynecology, Academic Hospital Cologne Weyertal, Germany

**Keywords:** Fibroid, pregnancy, gasless laparoscopy, abdominal pain, fibroid necrosis

## Abstract

The management of symptomatic uterine fibroids during pregnancy is a challenging situation. In some cases, surgical therapy can be required. Reports indicate that both laparotomy and laparoscopy are safe methods. However, laparoscopy is associated with less morbidity. This paper describes the case of a 31-year-old woman with a symptomatic uterine fibroid which was removed by gasless laparoscopy.

## Introduction

The prevalence of fibroids during pregnancy is estimated to be 10% (Selter et al. 2022). Despite a high prevalence of fibroids, there is limited information about the influence of fibroids on pregnancy (Selter et al. 2022). Fibroids during pregnancy grow mainly in the first trimester (Vitagliano et al., 2018; Ghosh et al., 2018). After the first trimester, the evidence is not clear, but there is a tendency for volume reduction in late pregnancy (Vitagliano et al., 2018). Most fibroids in pregnancy are asymptomatic, however some may cause symptoms depending on their number, size, and location (Vitale et al., 2015).

One of the most common symptoms of fibroids during pregnancy is pain (Deveer et al., 2012; Zaima and Ash, 2011). It can occur due to impaired vascularisation, usually in rapidly growing tumours, and lead to ischemia and necrotic degeneration of the fibroids (Lee et al., 2010). Another reason for severe abdominal pain is the torsion of pedunculated fibroids (Basso et al., 2017). Large fibroids can cause pain due to pressure on adjacent organs (Zaima and Ash, 2011). Fibroidectomy should be generally avoided during pregnancy because of the high risk of haemorrhagic complications, but in cases of severe abdominal pain that cannot be remedied with medical therapy, fibroidectomy may be required (Guidelines Committee of the Society of American Gastrointestinal and Endoscopic Surgeons and Yumi, 2008). There is some supportive evidence regarding gasless laparoscopy during pregnancy, which avoids the effects of carbon dioxide and high intraabdominal pressure (Shay et al., 2001; Schmidt et al., 2001; Akira et al., 1999; Curet, 2000). In the context of management, myomectomy is not commonly recommended during pregnancy because of concerns around potential consequences, including significant bleeding, uterine rupture, miscarriage, premature labour (Spyropoulou et al., 2020). The benefit of the myomectomy is that the fibroid-associated symptoms are quickly relieved. Myomectomy during pregnancy is associated with a miscarriage risk of 5.2% (Spyropoulou et al., 2020). The risk of late (34–37 weeks) and early (<34 weeks) preterm birth is reported to be 8.1% and 2.7%, respectively (Spyropoulou et al., 2020). One out of the 97 cases of myomectomy during pregnancy, with postsurgical myometrium necrosis followed by uterine rupture, was described in a recent review paper (Spyropoulou et al., 2020).

## Case Presentation

**Figure 1:**
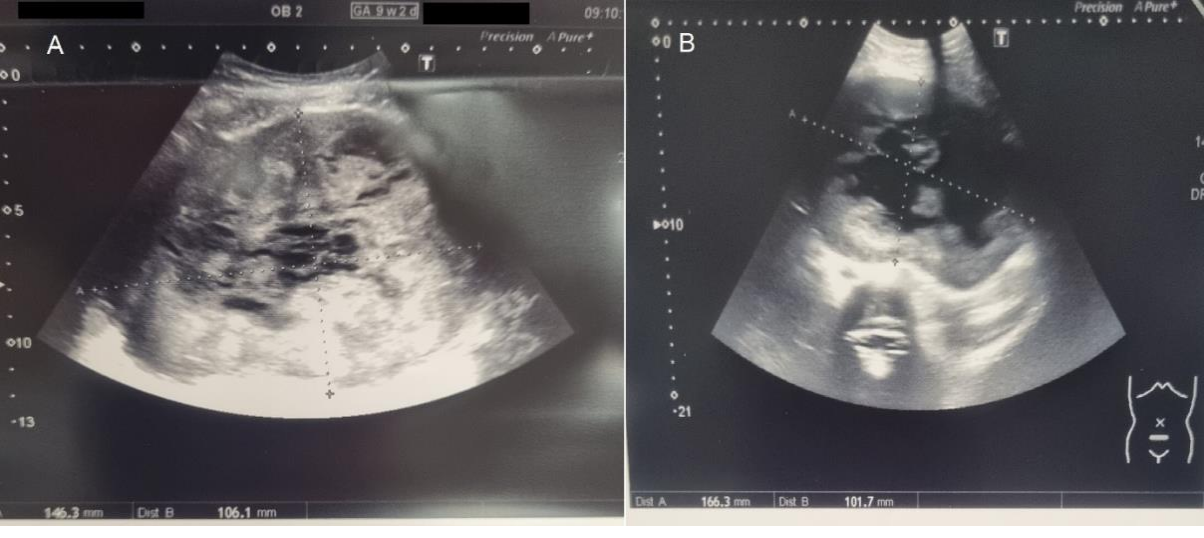
Sonographic examination of uterus fibroid. (A) Uterus fibroid at 9+2 weeks of pregnancy. (B) Uterus fibroid at 13 + 2 weeks of pregnancy.

**Figure 2:**
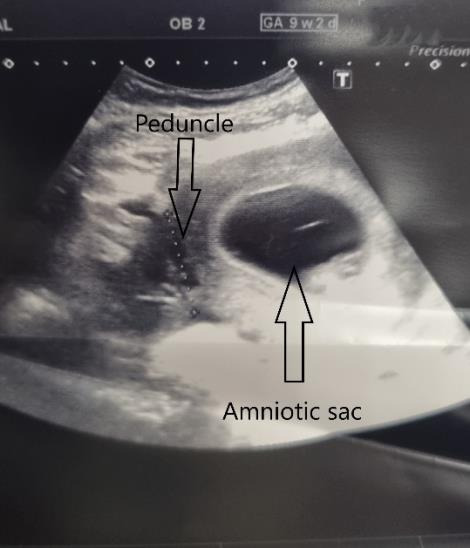
Peduncle of 4cm.

**Figure 3:**
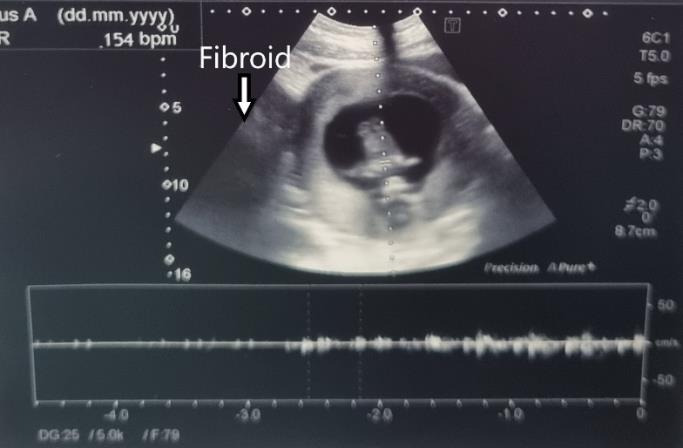
Intact pregnancy.

**Figure 4:**
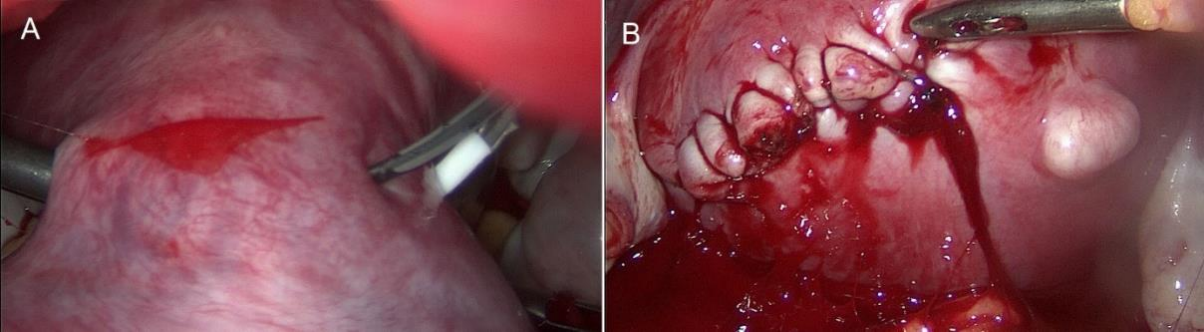
Laparoscopy. (A) Excision of the fibroid. (B) Suture.

**Figure 5:**
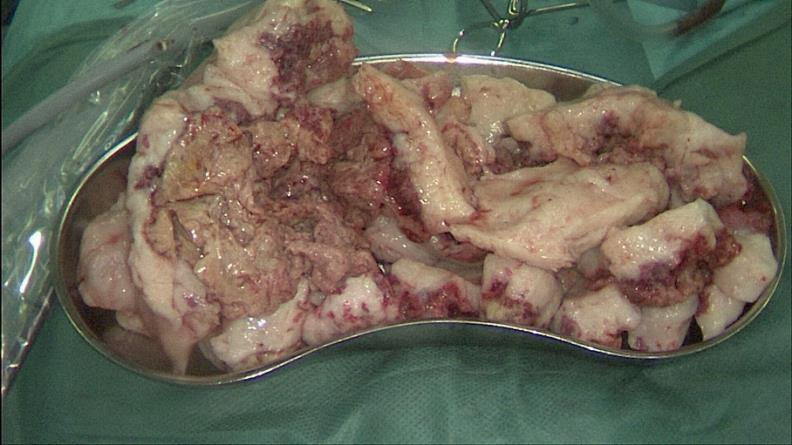
Fibroid after morcellement (810 g).

**Figure 6:**
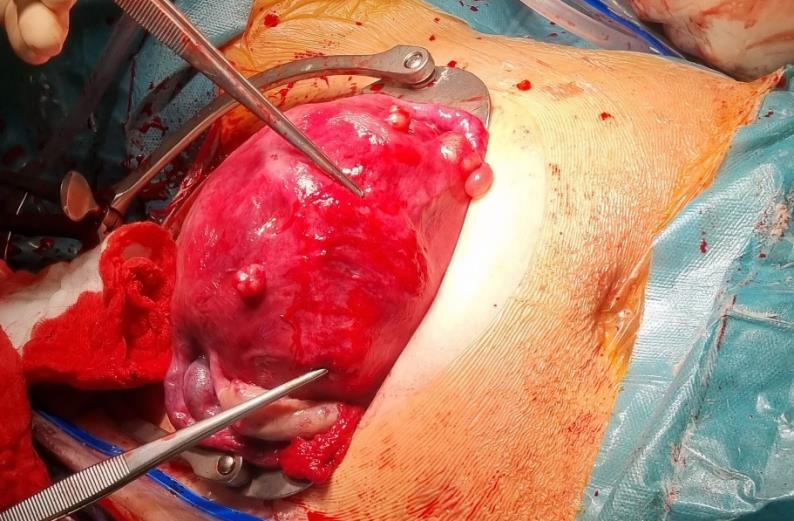
Caesarean section and the uterus scar 39 Weeks of pregnancy.

A 31-year-old GI woman attended our department at 9+2 weeks of pregnancy. Due to the presence of a sizable fibroid, there was intense abdominal pressure that caused the presentation. Prior to the pregnancy, a 7-cm fibroid was detected. The patient conceived naturally. During the initial weeks, she observed a growth in the size of the fibroid. She experienced a chronic elevation in intraabdominal pressure, leading to ongoing pain. No fever was reported. The levels of inflammatory markers were within the usual range. An abdominal tumour of substantial size was detected during a physical examination. Abdominal ultrasonography revealed an intact pregnancy and a large peduncled fibroid, measuring 146.3 x 106.1 mm (Figure 1A). A small amount of fluid accumulated within the fibroid, indicated the beginnings of tissue necrosis. The fibroid peduncle measured around 4 cm (Figure 2). Based on the symptoms observed, we recommended fibroid removal. Medical therapy (paracetamol, dihydrocodeine, NSAID (nonsteroidal anti-inflammatory drugs)) proved to be ineffective in this particular instance due to the fact that the symptoms were not induced by necrosis but rather by the dimensions of the fibroid. Surgical intervention was deemed necessary to alleviate the patient’s symptoms and address the underlying issue. The patient received detailed information regarding the potential risks and benefits of the procedure and provided consent to proceed to the surgical intervention. She was informed that the most favourable time for the surgery was during the beginning of the second trimester. Given the lack of notable symptoms aside from abdominal pressure, the surgery was scheduled for 4 weeks later. The repeat ultrasound scan detected a significant modification in the structure of the fibroid. The fibroid’s size has expanded to 166.3 x 101.7 mm, and there has been a significant augmentation in the quantity of fluid observed (Figure 1B). The pregnancy stayed unharmed (Figure 3). The surgery was carried out the next day. A laparoscopy was conducted using a gasless approach. First, the system for gasless laparoscopy was set up. A 3 cm incision was made in the umbilicus. The abdominal wall was elevated using the hooks. As there was no need for gas, there was no requirement to attach an optical trocar. On one side, this allowed for the prevention of injuries to the uterus and fibroid, as well as the avoidance of unnecessary repositioning of the trocar. On the other hand, it resulted in an improved visual perspective. Two 10 mm secondary trocars without ventils were inserted into the middle of the abdomen. The fibroid was excised using the Harmonic device (Figure 4A). As a result of the notable increase in blood vessels in the uterus during pregnancy, it was necessary to suture in order to halt the bleeding (Figure 4B). The 810 g fibroid was extracted via cord morcellation through the umbilical incision (Figure 5). As expected, the histopathological examination showed the fibroid tissue with necrotic changes. The patient was discharged from the hospital after a duration of two days and the abdominal ultrasound confirmed an ongoing pregnancy. The patient expressed a desire for a caesarean section. A caesarean section was conducted during the 39th week of pregnancy. A scar was discovered at the location where the fibroid was removed (Figure 6). The newborn exhibited normal development and was in good condition, with a height of 50 cm, a weight of 3060 grams, an APGAR score of 9/10/10, and a pH level of 7.22. The patient and child were discharged in a state of good health condition following a three- day period.

## Discussion

The majority of fibroids during pregnancy maintain their size and do not cause any symptoms, however some do cause symptoms dependent on their size, number and location (Topcu et al., 2015; Vilos et al., 2015). Recent data indicates that fibroids tend to have significant growth primarily within the initial 7 weeks of pregnancy. Following the first trimester, the evidence is not clear, but there is a tendency for volume reduction in late pregnancy (Coutinho et al., 2022). The occurrence of myomectomy during pregnancy is extremely rare, with the exception of cases of symptomatic fibroids, particularly in the first half of pregnancy (Vitale et al., 2015). Pain is the predominant symptom throughout pregnancy (Deveer et al., 2012). If pain arises from constant pressure caused by the substantial size of the fibroid, surgery may be required, although medical therapy can effectively address discomfort caused by necrosis or red degeneration (Phelan, 1995; Zaima and Ash, 2011). Conservative therapies includes rest, symptomatic analgesia, hydration, and assurance. Oral paracetamol and dihydrocodeine are safe, effective first-line analgesics. NSAIDs which have been shown to relieve pain, shorten hospital stay, and reduce re-admission, should be avoided after 34 weeks due to the risk of foetal nephropathy, premature ductus arteriosus closure, neonatal pulmonary hypertension, and platelet dysfunction. Opioids should only be used to treat acute pain not reduced by other painkillers; they have no use as maintenance therapy or prophylaxis (Zaima and Ash, 2011). Several studies have shown positive results when myomectomy was conducted during the second trimester of pregnancy, especially if the fibroid did not extend into the uterine cavity (Vitale et al., 2015). The second trimester is the optimal period for surgery due to several factors: the uterus is still small enough to not obstruct the surgical area, unlike in the third trimester; also, the potential risk of teratogenesis during the second trimester is quite low (Al-Fozan et al., 2002). Concerns about myomectomy include the higher chances of massive bleeding uterine atony, hysterectomy, pregnancy injury and/or pregnancy loss, and the development of adhesions between the uterus and the intestines after surgery (Vitale et al., 2015). Both laparoscopy and laparotomy are viable surgical procedures. The technical difficulty of laparoscopy is increased due to the enlarged uterus (Al-Fozan et al., 2002). Nevertheless, the majority of research in the existing literature indicate a predilection for using the laparoscopic procedure to treat fibroids during pregnancy. These studies also see laparoscopy as a secure and efficient strategy (Vitale et al., 2015).

Laparoscopy offers some advantages when compared to laparotomy. On one side, it is linked to decreased foetal depression as a result of decreased opioid utilisation during the postoperative phase. Laparoscopic surgery offers the benefit of requiring less manipulation of the uterus while still achieving sufficient exposure. This results in reduced uterine irritation, as well as a lower risk of spontaneous miscarriage, preterm labour, and premature birth (Al-Fozan et al., 2002).

### Laparoscopy during pregnancy: key points

When performing laparoscopy during pregnancy, it is important to consider the following points (Vitale et al., 2015; Al-Fozan et al., 2002; Ball et al., 2020):

- In the second half of pregnancy, patients should be positioned slightly towards their left side to support and alleviate impaired venous return
- Instruments should not be inserted into the cervix. - The secondary trocars should be placed higher than in nonpregnant patients. - To minimise the risk of maternal hypercapnia and foetal acidosis, the intraabdominal pressure should not exceed 10-15 mmHg when using carbon dioxide laparoscopy
- While manufacturer guidance varies, published evidence does not imply an increased risk of energy-related issues with any energy device, including monopolar, during pregnancy. When monopolar energy is used it is recommended that the return plate should not be placed such that the uterus is between the electrode and the plate
- Uterine mobilisation is limited and should be avoided

### Distinction between CO2 and gasless laparoscopies

In contrast to CO2 laparoscopy, during gasless laparoscopy, maternal and foetal acidosis as well as high intrabdominal pressure can be avoided (Al- Fozan et al., 2002; Römer et al., 2002). In addition, it is worth noting that a conventional closed laparoscopic procedure carries the risk of uterine injury due to the use of the Veress needle and/or optical trocar (Al-Fozan et al., 2002; Saccardi et al., 2015). An incidence of foetal loss that occurred at 21 weeks of pregnancy as a result of carbon dioxide insufflation into the amniotic cavity secondary to uterine damage caused by the Veress needle has been reported (Friedman et al., 2002). Furthermore, an additional benefit of the trocars without ventils is the capability of using conventional instruments in gasless laparoscopy (Römer et al., 2002).

## Conclusion

Generally, surgery in patients with fibroids should be avoided during pregnancy. However, in some cases with symptomatic fibroids, surgery may be required and should only be performed by a skilled surgeon. Laparoscopy has been proven to be a safe and successful procedure. To prevent the risks of maternal and foetal acidosis, excessive intraabdominal pressure, and uterus injury from a Veress needle or optical trocar, gasless laparoscopy may be a better option. Surgeons should be aware of the increased risk of bleeding, even if the fibroid is pedunculated, and therefore suturing may be required.
